# Abnormal connection between the posterior insula and the gastric network among patients with functional constipation

**DOI:** 10.3389/fnhum.2025.1624489

**Published:** 2025-10-17

**Authors:** Dehua Zha, Jiajing Chen, Yanyan Yang, Liang Zhang, Shaohua Tao, Wei Wang, Ming Li

**Affiliations:** ^1^Department of Anorectal Surgery, The First Affiliated Hospital of Anhui University of Chinese Medicine, Hefei, China; ^2^Department of Radiology, The First Affiliated Hospital of Anhui University of Chinese Medicine, Hefei, China

**Keywords:** functional constipation, resting-state functional magnetic resonance imaging, insula, gastric network, radiology

## Abstract

**Background:**

Functional constipation (FCon) is frequently accompanied by psychological disorders, implicating the interaction between the gastrointestinal symptom and brain dysfunction in FCon. Recent studies combining electrogastrogram and resting-state functional magnetic resonance imaging (fMRI) have reported a novel gastric network. Besides, the fMRI activity of the gastric network was also coupled with the insular fMRI signal. However, little is known about the connection between the gastric network and the insula in FCon.

**Methods:**

Based on rs-fMRI, functional connectivity (FC) using a large sample of 652 healthy subjects identified the insular cortex as the most closely linked to the gastric network. Then, seed-based FC and dynamic functional connectivity of the gastric network and the gastric-related insular cortex were calculated and compared in 35 patients with FCon and 36 healthy controls. Constipation symptoms were measured using the Patient Assessment of Constipation Symptom Scale (PAC-SYM) and the Wexner Constipation Scale. Their relationships with alterations in the gastric network-insula subregion were investigated.

**Results:**

The posterior insular cortex presented a strong connection with the gastric network with large-scale resting-state fMRI data sets of healthy participants. FCon patients had significantly decreased FC (t = −2.19, *p* = 0.032) in the left posterior insula and gastric network compared to healthy controls and were significantly negatively correlated with PAC-SYM (r = −0.407, *p* = 0.015) and Wexner Constipation Scale (r = −0.483, *p* = 0.003) scores.

**Conclusion:**

The abnormality of the connection between the posterior insula and the gastric network may be the prominent neuroimaging feature on FCon, which sheds light on a new perspective on the pathophysiology of FCon.

## 1 Introduction

Functional constipation (FCon) is one of the most common functional gastrointestinal disorders, which is a severe burden to patients, their families, and society (Simren et al., 2017). As a heterogeneous disorder, besides diverse gastrointestinal discomfort, a large number of FCon patients are accompanied by psychological disorders, mainly depression and anxiety (Hosseinzadeh et al., 2011). In addition, many patients with anxiety or depression frequently reported the problem of constipation (Ballou et al., 2019). These findings suggested a close relationship between brain dysfunction and FCon.

Indeed, the brain and the gastric system interact through direct and indirect connections (Mayer, 2011), contributing to normal feeding behavior as well as functional gastrointestinal disorders. Notably, altered gut-brain communication is implicated not only in functional GI disorders like irritable bowel syndrome (IBS), but also in neurodegenerative diseases such as Parkinson’s disease (Bellini et al., 2024; Bellini et al., 2023), where intestinal inflammation and barrier dysfunction are commonly observed. Recently, several studies have reported the existence of a novel brain network that shows phase synchronization with gastric activity, called the “gastric network” (Rebollo et al., 2018; Levakov et al., 2023). This network consists of several sensory and motor cortical areas whose resting-state activities are coupled explicitly with the stomach’s rhythmic activity measured with an electrogastrogram (ECG). The resting-state brain activities in previous gastric-network studies were mainly recorded using resting-state functional magnetic resonance imaging (rs-fMRI), a measure of spontaneous cerebral activities in blood oxygenation level-dependent signals without the constraints of the task-dependent paradigm. Although previous studies with rs-fMRI have demonstrated abnormal neural activities among the FCon patients (Yin et al., 2021; Cai et al., 2024), there are few studies focusing on the gastric network in the FCon patients so far.

While functional constipation is clinically characterized as a disorder of colonic function, the rationale for investigating a ‘gastric’ network in this context is grounded in the integrated nature of the gut-brain axis. The brain does not process signals from individual visceral organs in complete isolation. Instead, it often relies on centralized networks to regulate overall gastrointestinal homeostasis. The gastric network, identified by its synchronization with the rhythmic activity of the stomach, extends beyond gastric-specific functions. It encompasses key regions involved in somatosensory integration (e.g., postcentral gyrus), motor planning (e.g., supplementary motor area), and interoceptive awareness (e.g., cingulate cortex) (Rebollo et al., 2018; Rebollo and Tallon-Baudry, 2022). These functions are highly relevant to the pathophysiology of FCon, which involves altered visceral sensitivity, impaired sensorimotor coordination during defecation, and dysfunctional interoceptive processing of colonic signals (Mayer et al., 2022; Zhu et al., 2016). Therefore, we hypothesize that this network serves as a broader central hub for processing ascending visceral signals, including those from the colon. An abnormality within this network could disrupt the normal interpretation of colonic content and the generation of defecation urges, thereby contributing to the symptoms of FCon. Studying its interaction with the insula, a central interoceptive node, allows us to probe the integrity of this critical gut-brain communication pathway in a novel way.

The network partially overlaps with autonomic regulatory regions, regulates visceral activities, and plays a vital role in the autonomic effects (Rebollo and Tallon-Baudry, 2022). Indeed, plenty of evidence indicates that FCon patients tend to have impaired interoception (Mayer et al., 2022). The insula is generally regarded as the central cortical node in the interoceptive system and activated when individuals consciously focus on their interoceptive states, such as heartbeat and gastrointestinal sensations (Craig, 2002; Simmons et al., 2013). Considering its role in visceroception and close relationship with functional gastrointestinal disorder, we believe the altered activity between the gastric network and insula cortex in FCon patients.

However, the insular cortex is not included in the gastric network. The high heterogeneity of insular structures and functions may explain this incongruity. Several studies suggested that the insula is a heterogeneous cortical region composed of multiple structurally and functionally distinct subregions (Cauda et al., 2012; Sheffield et al., 2021). Specifically, the posterior insula is more linked with somatosensation (Stephani et al., 2011; Uddin et al., 2014). Intriguingly, Rebollo et al. (2018) reveal modest coupling of the posterior insula to several nodes of the gastric network. Hence, it is crucial to identify the insular voxel coupled with the gastric network. Recently, the coupling symptoms-related networks were identified using normative functional connectome data from large-scale resting-state fMRI data sets of healthy participants (Fox, 2018). This technique may be helpful for determining the insular cortex, showing close coupling with the gastric network.

Given the functional heterogeneity of the insula, it is crucial to identify the specific subregion that is most functionally coupled to the gastric network. While previous studies in FCon have reported alterations in the anterior insula, which is more associated with affective processing (Cauda et al., 2012; Uddin et al., 2014), our investigation is specifically motivated by evidence linking the posterior insula to primary interoceptive and somatosensory functions (Stephani et al., 2011; Uddin et al., 2014), and notably, by the work of Rebollo et al. (2018) which demonstrated a direct functional coupling between the posterior insula and the gastric network. Therefore, in the present study, we first aimed to identify the insular cortex closely linked to the gastric network based on the human connectome from a large sample of healthy cohort. Then, the connectivity between the gastric network and the gastric-related insula would be compared between FCon patients and matched healthy controls.

## 2 Materials and methods

### 2.1 Participants

This study included thirty-five FCon patients who were enrolled at the First Affiliated Hospital of Anhui University of Traditional Chinese Medicine in Hefei, China. All patients were diagnosed by a trained and experienced gastroenterologist according to the Rome IV criteria. The following were the exclusion criteria: (A) past or present neurological illnesses; (B) comorbidity with substance abuse, schizophrenia and other mental disorders; (C) suffering from congenital giant colon, redundant sigmoid colon, pelvic floor muscle relaxation and other gastrointestinal disorders; (D) head movements over 3 mm in fMRI scans; (E) additional contraindications to MRI. Besides, thirty-six age-matched healthy controls (HCs) were recruited through advertisements and matched to the FCon in terms of age and gender. Meanwhile, the large-scale resting-state fMRI data sets of healthy participants were used to obtain normative functional connectivity data based on our previous study (Bai et al., 2019).

The research was supported by the Anhui Medical University Ethics Committee and the Anhui University of Traditional Chinese MedicineEthics Committee. All participants signed an informed consent prior to enrollments.

### 2.2 Clinical evaluation

We evaluated the severity of patients’ constipation symptoms by the Wexner constipation scores scale and the Patient Assessment of Constipation Symptom Scale (PAC-SYM). In addition, we briefly evaluated the anxiety and depression levels using the Self-rating Anxiety Scale (SAS) and the Self-rating Depression Scale (SDS).

### 2.3 Neuroimaging data acquisition

The scanned images of the participants were acquired at the First Affiliated Hospital of Anhui University of Traditional Chinese Medicine, Hefei, Anhui Province. Resting-state MRI images were performed on a 3.0 T MRI scanner (Discovery GE750w) with an 8-channel head coil. Resting-state MRI was conducted with participants remaining awake and eyes closed using the following parameters: TR = 2,000 ms; TE = 22.5 ms; flip angle = 30; matrixsize = 64 × 64, field of view = 220 × 220 mm; slice thickness = 4 mm;33 continuous slices (one voxel = 3.4 × 3.4 × 4.6 mm). A T1-weightedanatomical image with 188 slices was also acquired for each patient to elucidate further and discard gross radiological alterations (TR = 8.676 ms; TE = 3.184 ms; inversion time = 800 ms; flip angle = 8; field of view = 256 × 256 mm; slice thickness = 1 mm; voxel size = 1 × 1 × 1 mm).

### 2.4 Functional data preprocessing

Preprocessing of the resting-state fMRI was conducted with Data Processing Assistant for Resting-State Functional MR Imaging (DPARSF) (Chao-Gan and Yu-Feng, 2010), a software based on the Statistical Parametric Mapping Software^1^ and the Resting-State Functional MR Imaging Toolkit.^2^ The processing steps we carried out for participants were as follows: discarding the first 10 volumes to exclude the impact of unsteady longitudinal magnetization, slicing time corrected, realigned, normalized to Montreal Neurological Institute (MNI) template, nuisance regressors with 24 Friston motor parameters, white matter high signal, cerebrospinal fluid and global signals, smoothed using a Gaussian kernel of 4 mm full-width at half-maximum and filtered using a temporal band-pass (0.01–0.1 Hz).

### 2.5 Definition of gastric network

**Figure 1:**
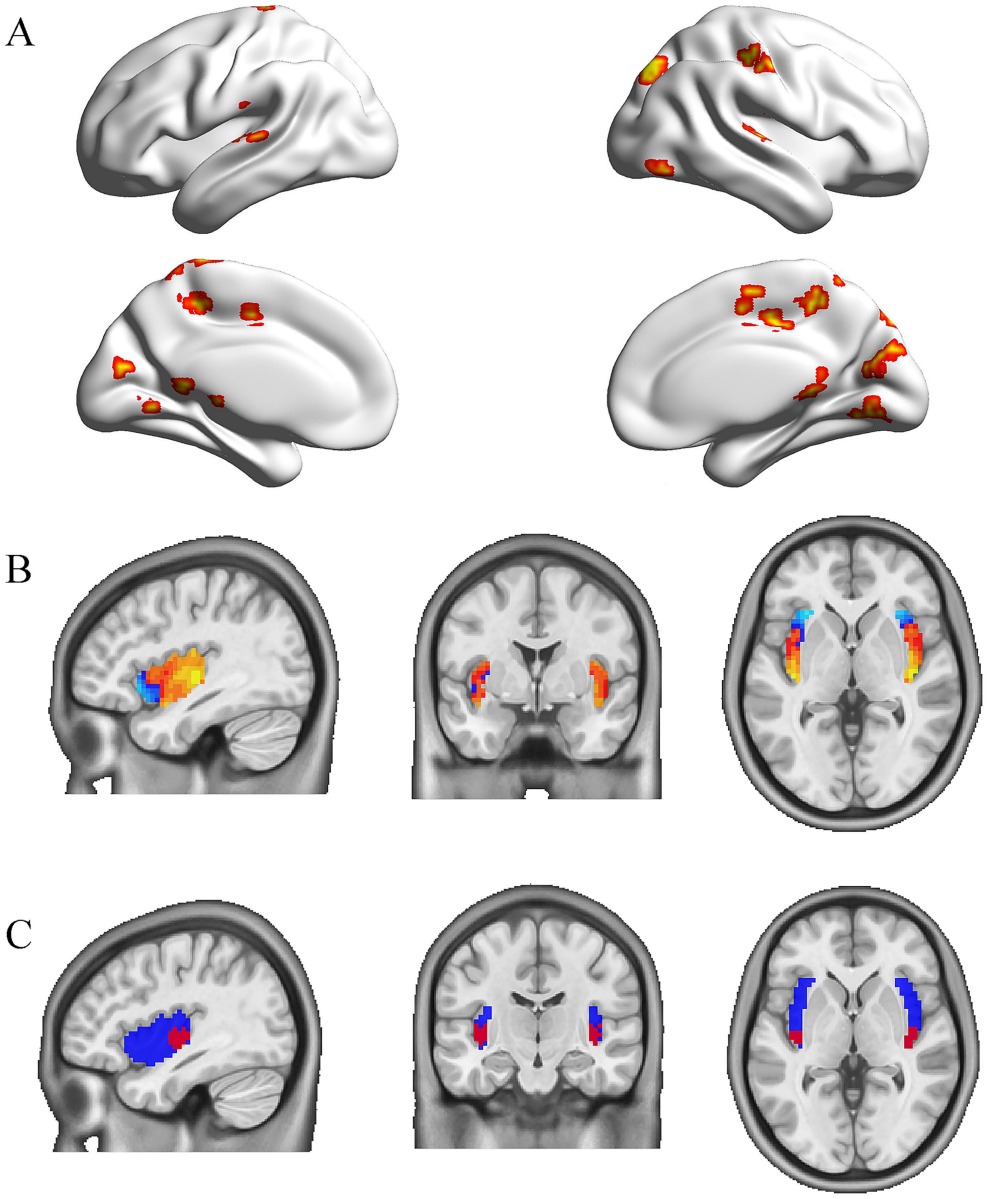
The gastric network and the subregion of the insula coupled to it. **(A)** The mask of the gastric network from the study by Rebollo et al. **(B)** Peak values of the insula coupled to the gastric network based on rs-fMRI data obtained from 652 healthy individuals. **(C)** Bilateral posterior insula masks (red) for 8 mm radius spheres in the whole insula (blue).

The gastric network reported by Rebollowas is composed of voxels with significant gastric-Bold phase synchronization obtained using a cluster randomization procedure, forming a threshold of *p* < 0.005 (one-sided) and all clusters Montecarlo *p* < 0.025 (one-sided).^3^ This network engaged subcortical and cortical regions, including the postcentral gyrus, superior temporal gyrus, supplementary motor area, cingulate gyrus, precuneus, and other regions (Figure 1A).

### 2.6 Definition of gastric-related insular cortex

The mask for the whole insula was taken from the Neurovault database and set as 20%.^4^ Given that Rebollo et al. (2018) observed modest coupling of the posterior insula with gastric-rhythm regions, we specifically targeted this subregion for its established role in interoception and visceral sensation. The mean gastric network signaling was correlated with whole insula voxels using Pearson correlation analysis on the rs-fMRI data from 652 healthy controls. These data were obtained from a previously published study by Bai et al. (2019) with written permission for use in the present analysis. All data had undergone consistent preprocessing pipelines, ensuring comparability with our acquired patient data. Then, one-sample *t*-tests were conducted for these functional connectivity maps (Fisher’s r-to-z transformation). The t-map showed the connectivity pattern of the gastric-derived insula map. Within this specificity network, we identified two insula clusters (right and left posterior insula, peak coordinates 39, −15,0 and −39, −18,0) with the highest functional connectivity (FC) values (Figure 1B). Then, the bilateral spheres with an 8 mm radius were used as insula subregions in the whole insula mask (Figure 1C).

### 2.7 Seed-based functional connectivity analysis

The gastric network and the gastric-related insular cortex described above were used as seed regions to calculate their functional connectivity. For each participant, Pearson’s correlation coefficients were calculated between the two seeds for the resting-state BOLD signal time series, and Fisher’s r-z transform was applied.

### 2.8 Seed-based dynamic functional connectivity analysis

The gastric network and the insula subregions described above were used as seed regions to calculate their dynamic functional connectivity (dFC). The flexible least squares approach was applied to assess the dFC of each participant using the DynamicBC toolbox.^5^ Then, we investigated the temporal variability by measuring the standard deviation and variance of the dFC maps.

### 2.9 Statistical analysis

Age was compared between the FCon and HCs groups using the independent samples *t*-test. The values of FC and dFC between the gastric network and gastric-related insular cortex were extracted, and independent samples *t*-tests were conducted to compare the differences between the two groups. Significance was defined as *p* < 0.05. Moreover, Spearman correlation coefficients were employed to investigate the relationship between FC values and dFC values with constipation levels, anxiety levels, and depressive levels in FCon patients. The significant threshold was set at 0.05 (two-tailed).

## 3 Results

### 3.1 Demographic and clinical characteristics

Demographic and clinical information about the participants was listed in Table 1, which included 35 FCon and 36 HCs. No significant differences were observed in the age (t = −0.23, *p* = 0.821) and gender (*χ*^2^ = 0.05, *p* = 0.832) within the two groups.

**Table 1:** Demographic and clinical information.

	Patients	Controls	*χ*^2^/T	*p*-value
Sample size (*n*)	35	36		
Age (years)	48.91 (15.61)	49.75 (15.44)	−0.23^a^	0.821
Gender (male/female)	8/27	9/27	0.05^b^	0.832
Wexner	15.63 (4.97)	NA		
PAC-SYM	18.74 (5.85)	NA		
SAS	32.89 (8.91)	NA		
SDS	40.57 (11.69)	NA		

PAC-SYM, Patient Assessment of Constipation Symptom Scale; SAS, Self-rating Anxiety Scale; SDS, Self-rating Depression Scale. Data are presented as the mean (standard deviation). ^a^*T*-value; ^b^Chi-square.

### 3.2 Group-level comparison in the functional connectivity

**Figure 2:**
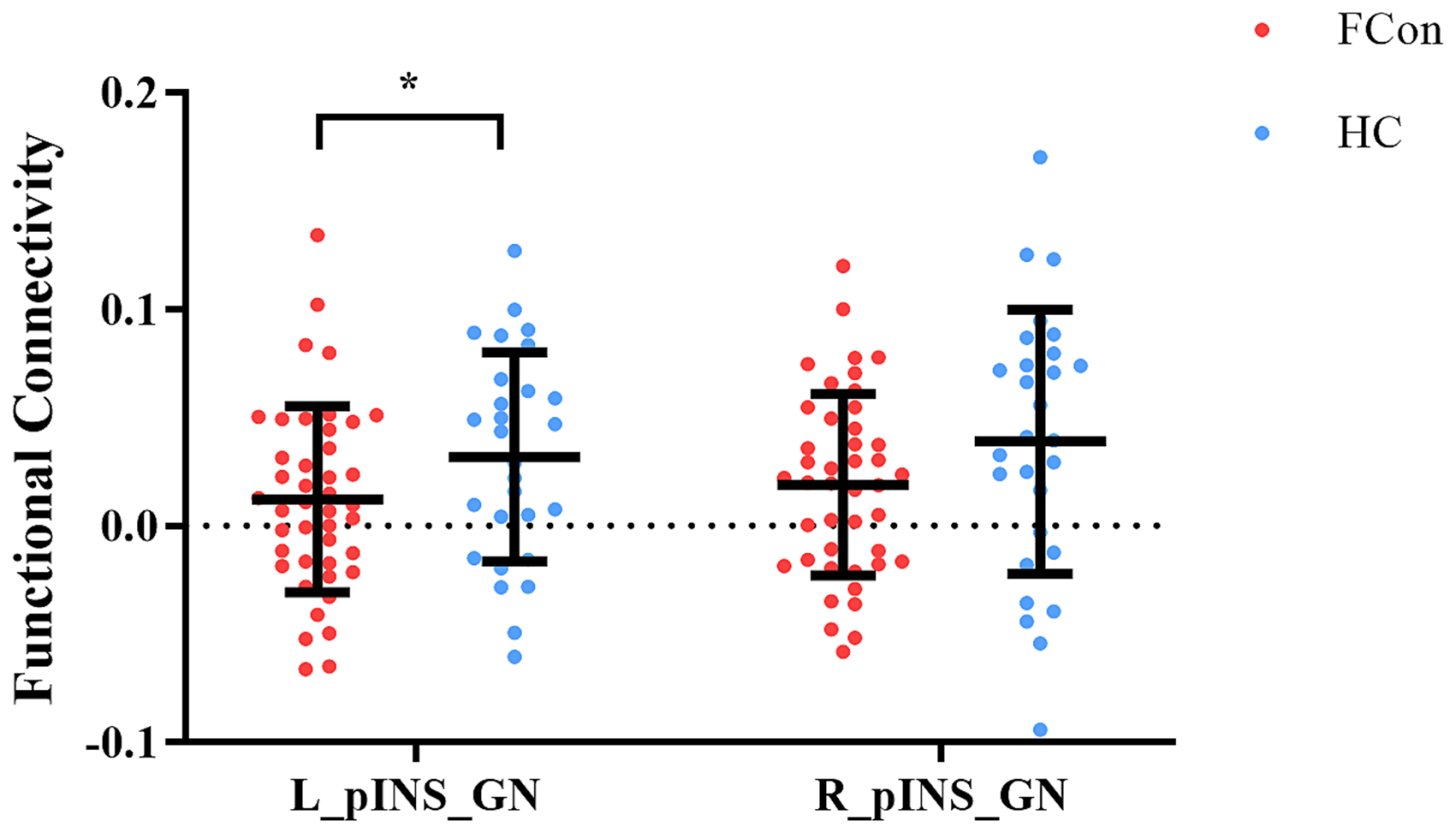
Differences in FC of pINS and GN in the two groups (FCon and HCs). Significantly lower FC of left pINS and GN in FCon compared to HCs. FC, functional connectivity; pINS, posterior insula; GN, gastric network; FCon, functional constipation; HCs, healthy controls; L, left; R, right. *p < 0.05.

The independent samples *t*-test showed that the mean values of FC for the left posterior insula and gastric network (t = −2.19, *p* = 0.032, Cohen’s d = 0.52) were significantly different in FCon and HCs groups, though this did not survive Bonferroni correction for two comparisons (adjusted *α* = 0.025). The right posterior insula showed a non-significant trend in the same direction (t = −1.54, *p* = 0.129, Cohen’s d = 0.37) (Figure 2). This lateralized effect suggests a potential left-hemisphere dominance in visceral interoceptive processing in FCon, consistent with some prior literature on gut-brain lateralization.

### 3.3 Group-level comparison in the dynamic functional connectivity

**Figure 3:**
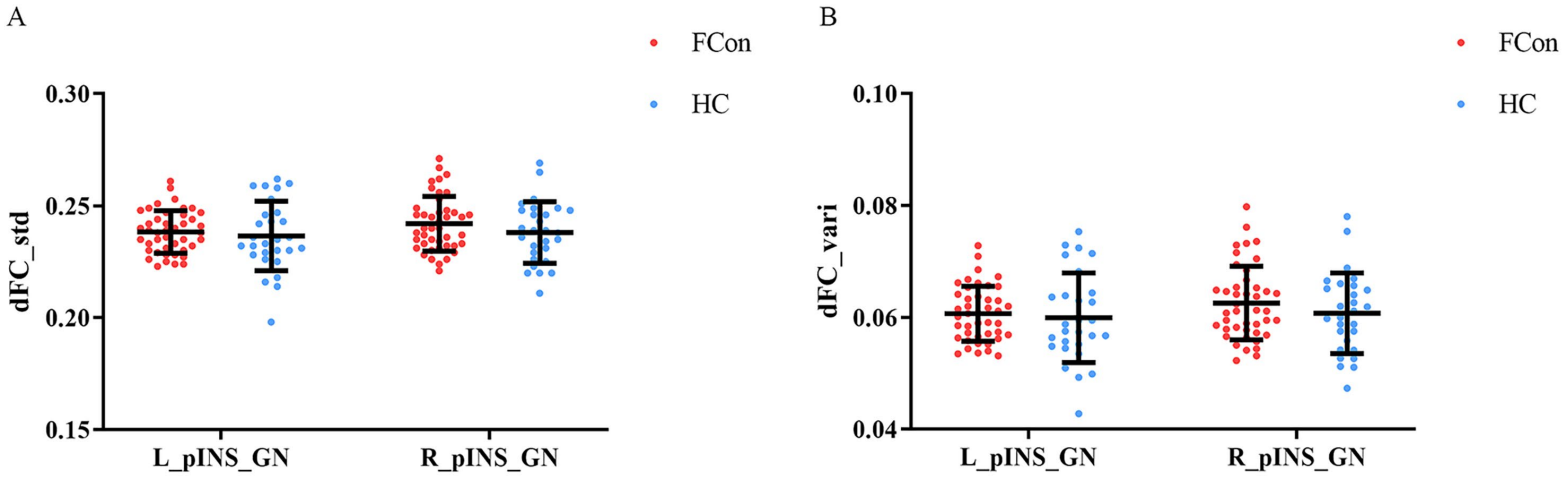
Differences in dFC of pINS and GN in the two groups (FCon and HCs). **(A)** No significant differences in standard deviation dFC values of left pINS-GN and right pINS-GN in FCon compared to HCs. **(B)** No significant differences in variance dFC values of left pINS-GN and right pINS-GN in FCon compared to HCs. dFC, dynamic functional connectivity; pINS, posterior insula; GN, gastric network; FCon, functional constipation; HCs, healthy controls; L, left; R, right.

No significant differences were found in the temporal variability of insula–gastric network connectivity, as measured by standard deviation or variance of dFC. Independent samples *t*-tests showed that the standard deviation values (t = −0.92, *p* = 0.364) and variance values (t = −1.08, *p* = 0.287) of dFC for the left posterior insula and gastric network were not significantly different between FCon and HCs groups. Analogously, independent samples *t*-tests showed that the standard deviation values (t = −0.03, *p* = 0.974) and variance values (t = −0.21, *p* = 0.839) of dFC for the right posterior insula and gastric network were not significantly different in the two groups (Figure 3).

### 3.4 Correlation analyses

**Figure 4:**
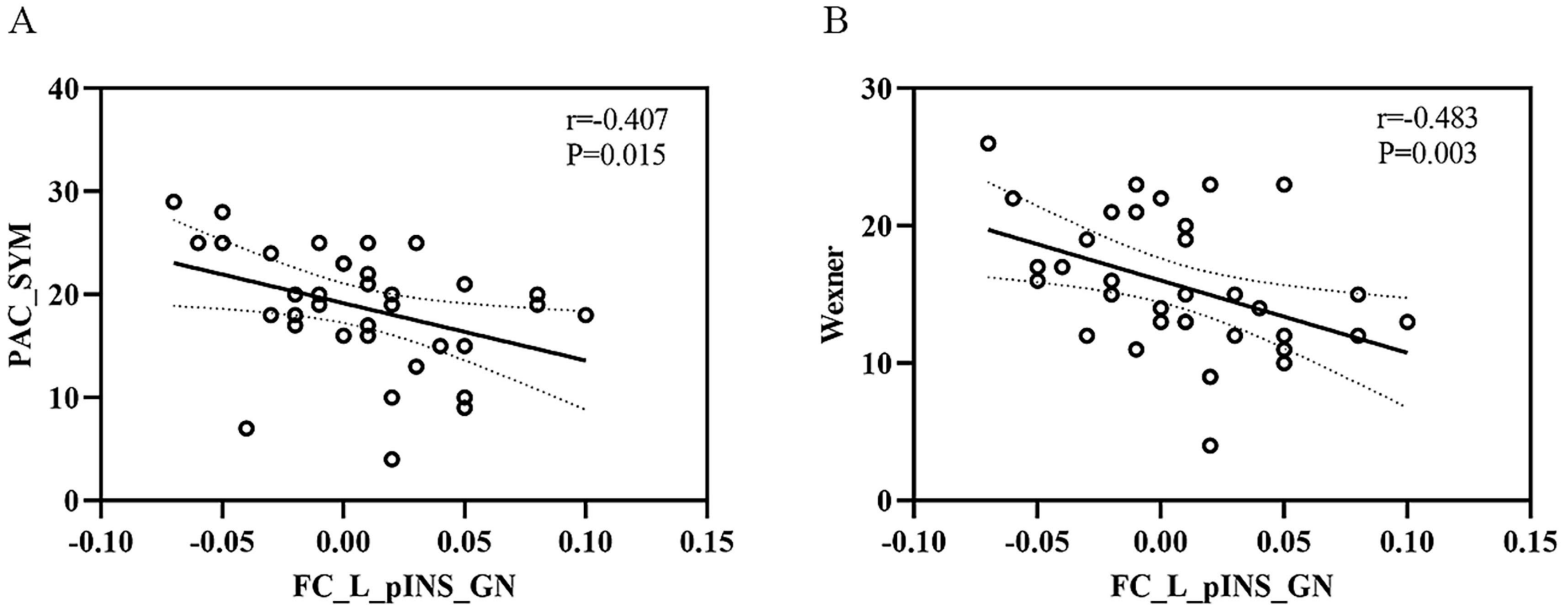
The relationship between FC values of left pINS-GN and clinical symptoms in FCon. **(A)** Significant negative correlation between FC values of left pINS-GN and PAC-SYM scores in FCon. **(B)** Significant negative correlation between FC values of left pINS-GN and Wexner constipation scores in FCon. FC, functional connectivity; pINS, posterior insula; GN, gastric network; FCon, functional constipation; PAC-SYM, Patient Assessment of Constipation Symptom Scale; L, left.

Lower FC values in the left posterior insula–gastric network were significantly correlated with worse constipation symptoms (higher PAC-SYM and Wexner scores), but not with anxiety or depression scores. We identified significant negative correlations between FC values in the left posterior insula-gastric network and PAC-SYM scores (r = −0.407, *p* = 0.015) as well as Wexner constipation scores (r = −0.483, *p* = 0.003) in FCon patients. In addition, no significant correlations between FC values of the left posterior insula-gastric network and SAS scores (r = −0.094, *p* = 0.592) as well as SDS scores (r = −0.113, *p* = 0.519) were observed in FCon patients (Figure 4).

## 4 Discussion

In the present study, we aimed to investigate the connection patterns between the gastric network and the insula subregion and their relation to clinical symptoms in FCon patients. The novel work is the identification of gastric-related insular cortex with large-scale resting-state fMRI data sets of healthy participants. Based on this finding, we found lower functional connectivity of the left posterior insula and the gastric network among FCon patients, and the decreased functional connectivity was significantly negatively correlated with constipation symptoms. Our findings indicated that FCon had abnormal functional coupling between the posterior insula and the gastric network, which is a prominent feature.

Previous neuroimaging studies have reported that FCon shows significant brain functional and structural alterations involving areas of sensory processing, motor control, self-referential processing, and emotional modulation (Yin et al., 2021; Liu et al., 2021). In resting-state functional MRI, Zhu et al. (2016) found functional abnormalities in the precentral gyrus, supplementary motor area (SMA), and the cingulate cortex in FCon patients using the amplitude of low frequency fluctuation (ALFF) metric. Subsequently, a number of rs-fMRI studies demonstrated functional impairments in the postcentral gyrus, precuneus, and other areas (Zhang et al., 2022). Furthermore, several structural MRI studies revealed that FCon patients showed altered cortical morphometry in the SMA, posterior cingulate gyrus, and precuneus area (Hu et al., 2020). These findings may partly illuminate the potential neural mechanisms in FCon. Strikingly, we noticed that the brain regions with significant abnormalities among FCon patients in previous studies highly overlapped with those involved in the gastric network, and thus this could support our hypothesis of dysfunction of the entire gastric network in FCon patients. Moreover, few studies have explored how FCon patients’ gastric network functions. This weakened coupling may reflect impaired interoceptive awareness in FCon, potentially leading to blunted defecation urges or dysregulated gut motility. Furthermore, recent evidence suggests that gut inflammation and dysbiosis may disrupt gut-brain signaling, as seen in Parkinson’s disease (Bellini et al., 2024; Bellini et al., 2023), which could similarly apply to FCon.

The insula is engaged in integrating and processing information from interoceptive sources and plays an essential role in coordinating proper emotional, behavioural, and visceral responses (Uddin et al., 2017; Benarroch, 2019). Stephani et al. (2011) showed that electrical stimulation of the insula cortex elicited visceral sensory reactions including abdominal sensations, gastric vibrations, and emesis. Previous studies found abnormal baseline brain activity in the insula and a negative correlation between ALFF of the insula and defecation difficulties for FCon patients (Zhu et al., 2016). Meanwhile, structural MRI studies showed that FCon patients had decreased grey matter volume in the insula, which was negatively associated with abdominal symptoms (Jia et al., 2022). A systematic review of fMRI revealed increased ALFF value of the insula following acupuncture treatment in FCon (Wang et al., 2024). Furthermore, prior research has shown that the posterior insula was coupled to the entire gastric network (Rebollo et al., 2018). Consistent with this, we identified that the posterior insular cortex presented a strong connection with the gastric network with large-scale resting-state fMRI data sets of healthy participants. Extending these observations, our key finding is that functional coupling between the posterior insula and the gastric network is significantly reduced in patients with FCon and is negatively correlated with constipation severity.

Our finding of altered connectivity in the posterior insula presents an interesting contrast to some previous FCon studies that emphasized alterations in the anterior insula (Zhu et al., 2016; Jia et al., 2022). This discrepancy, however, may reveal complementary rather than contradictory pathophysiological mechanisms. The anterior insula is predominantly involved in the affective appraisal of visceral sensations, which aligns with the psychological comorbidities seen in FCon. In contrast, the posterior insula serves as a primary cortex for receiving and processing visceral sensory inputs (Stephani et al., 2011; Uddin et al., 2014). Therefore, the hypo-connectivity we observed may reflect a more fundamental deficit in the initial transmission and perception of gut signals, potentially leading to a blunted defecation urge. We speculate that this aberrant connection in FCon alters defecation control primarily by disrupting basic internal information perception. Thus, abnormalities in the anterior and posterior insula may represent dysfunction at different levels of a hierarchical interoceptive pathway in FCon, with our results highlighting a critical impairment in the primary sensory processing stage.

Several limitations of this study deserve discussion. Firstly, the relatively small sample size of the FCon constrained statistical power, and larger sample sizes could be required to replicate the current experiments in the future. Second, prior studies showed gender distinctions in resting-state functional connectivity for FCon (Jin et al., 2019), so the male to female ratio might impact our findings. Third, we did not systematically control for or record the use of medications, including laxatives and nutraceuticals, among the patient cohort. The potential effects of these substances on brain function or symptom severity could represent a confounding factor that our study was not designed to address. Finally, this study used the rs-fMRI approach, but rs-fMRI could not accurately reflect the event’s relevance, and future experiments could employ task-state fMRI to investigate.

## 5 Conclusion

In conclusion, we found that patients with FCon displayed significantly reduced FC between the left posterior insula and the gastric network, and further discovered that this decreased connectivity was significantly negatively associated with constipation symptoms. These findings indicate prominent neuroimaging features on FCon, which shed light on a new perspective on the pathophysiology of FCon.

## Data Availability

The original contributions presented in the study are included in the article/supplementary material, further inquiries can be directed to the corresponding authors.

## Footnotes

^1^ www.fil.ion.ucl.ac.uk/spm

^2^ http://www.restfmri.net

^3^ https://neurovault.org/images/51888/

^4^ https://neurovault.org/collections/6208/

^5^ www.restfmri.net/forum/DynamicBC
